# Micro–nano scale motion control of light-driven morphing pillars for biointerfacing†

**DOI:** 10.1039/d4nr04659e

**Published:** 2025-06-10

**Authors:** Ziyu Gao, Valeria Criscuolo, Fabio Formiggini, Ilaria De Martino, Daniela Perna, Velia Siciliano, Francesca Santoro

**Affiliations:** a Center for Advanced Biomaterials for Healthcare, Istituto Italiano di Tecnologia Naples Italy f.santoro@fz-juelich.de; b Institute of Biological Information Processing – Bioelectronics, IBI-3, Forschungszentrum Jülich Jülich Germany; c Neuroelectronic Interfaces, Faculty of Electrical Engineering and Information Technology, RWTH Aachen Aachen Germany

## Abstract

Morphic biomaterials have been widely utilized as cell instructive platforms to regulate biological functions of cells and tissues. Here, light can be exploited to tune the mechanical response of these materials (*e.g.*, azobenzene-based polymers) to reshape and induce dimensionality changes (*e.g.*, from 2D to 3D geometries). In fact, 3D systems better replicate the cell–tissue environment and its continuous conformational changes. In this work, we present a 3D pillar-based platform capable of undergoing reshaping and dislocation. These properties can be precisely controlled at the micro–nano scale through characteristic light irradiation parameters such as intensity and exposure time. In this work, light-driven deformation resulting in elongation, polarization, and substructure generation (*i.e.*, nanobump-like structures) was achieved using low-intensity (5%) light exposure. Furthermore, deformation in sliding and rotation was obtained with high laser intensity (20–100%, % of 10 mW input), based on which, a photo-induced deformation model was proposed for azobenzene-based polymers. Additionally, the biocompatibility of the azopolymer-based pillars was proven as well as their potential to locally reshape and induce real-time cell response.

## Abstract 2

This study presents a light-responsive platform based on azobenzene polymer micropillars that enables precise, reversible micro-nanoscale deformations *via* laser stimulation, demonstrating biocompatibility and potential for real-time cell modulation.

## Graphical abstract

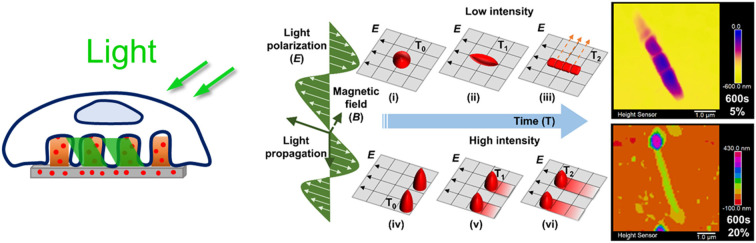

## 1 Introduction

The development of biomedical devices that can actively respond to native tissues and cell movements have a significant role in the field of accurate medical technology.^1^ Attention has been generated in the field of morphing materials, *e.g.* flexible electronics,^2^ which could adapt to living tissue movement and biosignalling tracking. However, most of the currently available devices are passive systems, and are not able to actively couple with cell movement or stimulate native tissues. Biomimetic devices that can behave like cells to provide dynamic environments to emulate cell functions are therefore required.^3,4^ To this end, the design and engineering of dynamic cell–material interfaces could enable active regulation of biointerfaces through mechanical coupling, including integration, adaptation, movement, *etc*.^1,2,5^

Dynamic interfaces can be obtained with stimuli-responsive materials triggered, for example, by electricity, temperature, and light.^6,7^ Here, light-responsive polymers have been widely used for biological cell interfacing, thanks to their high controllability and low invasiveness.^3,6^ In particular, light-driven stimuli-responsive polymers have been widely used as cell-instructive biomaterials to interface cells and tissues and impart biomechanical stimuli to regulate certain cellular processes. In fact, these platforms demonstrated reliable morphing properties, which allowed shape change, and were also exploited for cell culture. For instance, in surface relief grating (SRG) techniques, patterns can be created on a micrometer scale, which can also regulate cell fate.^8–11^

Furthermore, the high resolution and control provided by the laser beam polarization and intensity should strongly affect the polarization direction, resulting in a defined surface pattern and generating a local force. In fact, the induced mass transport can support bullet-like and high aspect ratio patterns.^10–12^

Azobenzene-based polymer materials, in particular, showed reliable morphological controllability in response to light.^13,14^ Protruding vertical and 3D structures have been successfully employed for cell interfacing, achieving local cytoskeleton stimulation and response.^15–17^ Moreover, the recent report on the photo-induced crawling phenomenon demonstrated light-driven crystal displacement.^18^ Using two different laser beams (365 nm and 465 nm) could generate an asymmetric force within individual patterned structures, leading them to crawl,^19^ although an optical-driven force could thin the crystals during the crawling due to photofluidization of the polymers.^20^ This phenomenon allows induction of an optical-field gradient force by applying high-intensity laser irradiation^11^ and paved the way for further control of photo-isomerizable materials to allow micro–nanoscale motion or multidimensional deformation at the functional biointerface, *e.g.* conductive substrates.^21^ In this work, we investigated the imprinting technique to mold poly(disperse red 1 methacrylate) (pDR1m) into protruding vertical structures. Simultaneous reshaping and dislocating were studied to achieve precise morphological control, and furthermore, potential regulation of biological cell activities.

The light-driven deformation of pDR1m micropillars was investigated *via* stimulation with a 555 nm single wavelength laser (linear polarized), considering the intensity- and time- dependent exposure. Ultimately, the controllable light-driven shaping and movement were achieved by applying low and high laser beam intensities, respectively. Furthermore, a biocompatibility assay confirmed the material capabilities of the interface with cells and provided localized stimulation and cytoskeleton regulation at single cell resolution. According to the light-driven control of both morphology and motion at the micro–nano scale, this work suggests reliable, optically based applications of pDR1m pillars in localized cell modulation, accurate medicine technology, and smart intelligent nanotechnologies, such as soft nanorobots.

## 2 Results and discussion

**Fig. 1:**
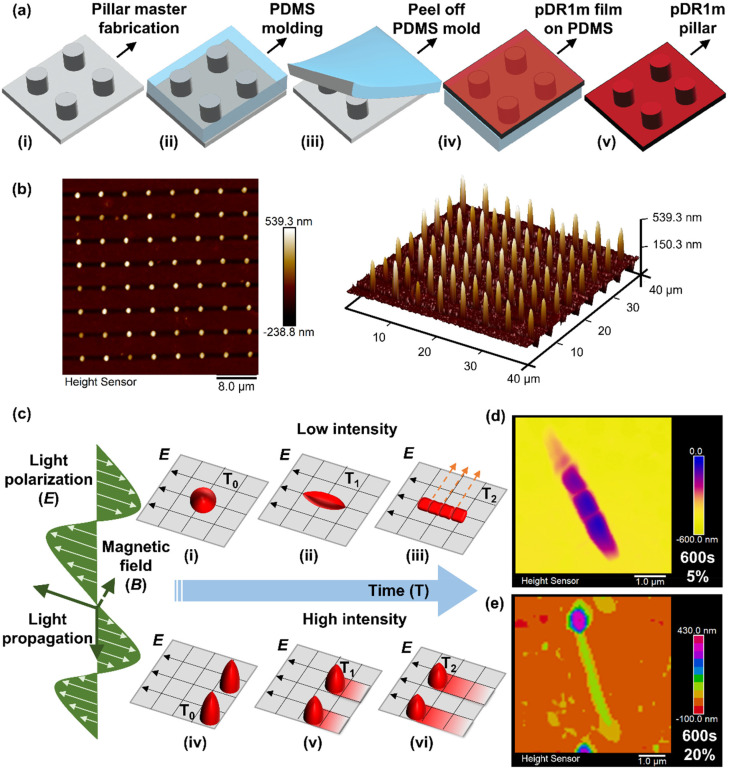
Schematic illustration of poly(disperse red 1 methacrylate) pillar array fabrication and light-driven morphing properties. (a) Fabrication process of pDR1m pillar arrays by the replica molding technique: (i) master fabrication by 2-photon lithography with an IP-DIP photoresist, (ii) soft PDMS mold fabrication by thermal curing at 80 °C for 4 hours, (iii) the PDMS mold with pillar size holes was peeled off from the master substrate, (iv) the pDR1m film was spin coated on a glass substrate and then flipped onto PDMS molds for imprinting at 150 °C overnight, (v) imprinted pDR1m pillar arrays were obtained by peeling off the PDMS molds. (b) Morphological illustration of pDR1m pillars by AFM in 2D (left) and 3D views (right). (c) Schematics of light-driven deformation and motion mechanisms of pDR1m pillars under various light conditions and time scales, according to light transportation parameters: propagation, polarization (*E*) and magnetic field (*B*). Pillar deformation illustration at low intensity from the initial status (c-i) to elongation (c-ii) and topographic re-orientation (c-iii), and pillar sliding behaviour at the laser polarization direction at high intensity from the initial position (c-iv) to further distances in time function (c-v and c-vi). Depicted AFM visualization of pillar deformation after 600 s of stimulation: topographic re-orientation under 5% laser intensity (d) and pillar sliding under 20% laser intensity. (e) Percentage of the total 10 mW input laser power.

The pDR1m pillars were fabricated by replica molding (Fig. 1a) as previously shown^15^ and are described in the Experimental section. Here, photoresist-based pillar arrays patterned by 2-photon lithography were used as a hard master (Fig. 1a, i) for the fabrication of a polydimethylsiloxane (PDMS) mold (Fig. 1a, ii and iii). To thermally imprint pillars into the pDR1m films, spin coated films were flipped onto soft PDMS molds and baked overnight in an oven at 150 °C (> glass transition temperature of pDR1m, Fig. 1a, iv); finally, pDR1m pillar arrays were obtained by peeling off the mold (Fig. 1a, v). Based on a master layout of 1 μm height, 1 μm diameter and 4 μm pitch-to-pitch distance (namely, H1D1P4, Fig. S1†), pDR1m pillar arrays with 459 ± 66 nm in height and 1.14 ± 0.04 μm in diameter were fabricated and measured by AFM (Fig. 1b).

To investigate the light-driven response of pDR1m pillars, a 555 nm laser with a total 10 mW input power was applied to stimulate pillars with different laser intensities and illumination times (Fig. 1c). As a result, after 0–600 s of laser stimulation, a stable pillar deformation was obtained at low exposure intensity (5%) outlining elongation, polarization, and sub-structure generation. All laser power percentages in this work are based on a total input power of 10 mW; unless otherwise stated, the actual output power was measured at the objective panel and is listed in Table S1.†

The final deformation of pillars was obtained after 600 s of light exposure at 5% laser beam intensity with the appearance of nanobump-like protruding structures (Fig. 1d), while continuous sliding and rotation were obtained at high intensity (20–100%) as shown in Fig. 1e. Optical images (Fig. S2 and 3†) and real-time video recordings (ESI, videos 1–3†) revealed continuous deformation of pDR1m pillar arrays while responding to the 555 nm laser at different intensities in real time.

**Fig. 2:**
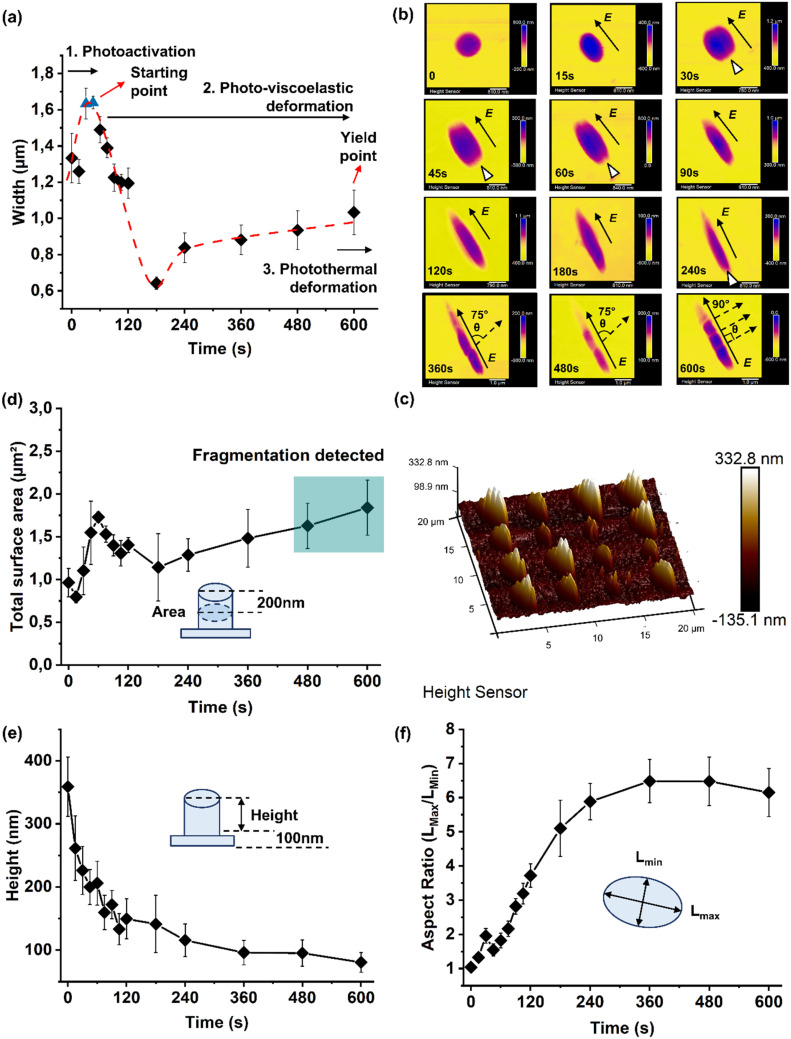
Poly(disperse red 1 methacrylate) pillar fabrication and light-driven deformation investigation under a 555 nm laser with 5% intensity at an illumination time from 0 to 600 s. (a) Width and the proposed photo-induced viscoelastic deformation mechanism for pDR1m pillars, laser intensity: percentage of the total 10 mW input power. (b) Light-driven deformation tracking on pDR1m pillar array morphologies stimulated by a 5% intensity laser from the initial status up to 600 s. *E* indicates the polarization direction and *θ* indicates the re-orientation angle. (c) Surface area analysis (200 nm threshold) under 5% intensity, after different exposure time periods from 0 up to 600 s. (d) Collective morphological illustration of nanobump-like structures after 600 s of laser stimulation. (e) Height change quantification (100 nm threshold) and (f) aspect ratio (*L*_max_/*L*_min_) tracking of pDR1m pillar array deformation. Laser intensity: 5% of the total 10 mW input power.

A time-dependent light stimulation and morphological analysis were carried out to monitor the deformation dynamicity. A low laser beam power (5%) was first investigated. As presented in Fig. 2a, a significant increase of pillar width was observed from the initial width of 1.33 ± 0.14 μm to 1.63 ± 0.09 μm after 30 s of light exposure, reaching a width plateau (blue triangles). After this, the pillar width begins to decrease, reverting to its initial value (1.23 ± 0.08 μm) after 90 s of light stimulation. A flat conformation was recorded from 90 s to 120 s of exposure as a result of the saturated chromophores’ activation and the completion of the azo-molecule structural motion.^10,22^ The width continued to decrease from 120 s to 180 s, followed by a slight increase from 240 s to 600 s of light exposure (Fig. 2a).

Building on the mechanical deformation process of polymer materials, a photo-induced deformation model was proposed after analyzing the changes in pDR1m pillar width under 5% laser stimulation. This model reveals three distinct behaviors: photoactivation, photo-viscoelastic deformation, and photothermal deformation. Each behavior is marked by specific points, with the initial activation starting early in the process (between periods 1 and 2) and the yield point occurring slightly later (between periods 2 and 3), as illustrated in Fig. 2a. In the photo-viscoelastic deformation phase, two distinct behaviors are theorized to occur due to the *trans*–*cis* transition of azobenzene molecules: chromophore isomerization and pillar domain motion. The motion of the azobenzene chromophores began after 30 s, when the width reached a plateau, followed by pillar domain motion with a decrease in width from 120 s, reaching the yield point of the viscoelastic deformation region at 600 s of light exposure. The potential reversibility of the photo-viscoelastic deformation occurring during this stage could depend not only on the light stimulation, but also on the polymer deposition and stimulation conditions.^20,24^

Continuous laser stimulation over 600 s (in the case of 5% laser stimulation) will induce photothermal deformation (photo-plastic), during which the morphological change is due to the thermal effect rather than the light-driven molecular response, resulting in thermal plastic deformation. The deformation that occurs in this period can no longer be reversed by the light source.

Furthermore, this light-driven deformation model was supported and defined with visible morphological changes. Identified deformations in elongation, polarization, and substructure generation (*i.e.*, nanobump-like structures) are shown in Fig. 2b and Fig. S5.† The pillars were found to stably deform with the direction of the linear laser polarization vector, *E*, consistent with other reports on azo-based films,^23^ colloids,^25^ nanoholes,^26^ and nanopillars.^13,26^

Moreover, the rotation of the pillar array along with different laser irradiation directions also induced re-shaping of the pillars, proving that this process is strongly dependent on light polarization (Fig. S6†).

Notably, the appearance of the *E* polarization line in the middle of the pillar structure after 30 s of illumination suggested that the azobenzene isomerization was initiated; in fact, here, only the chromophores that had a transition of the dipole moment with a direction parallel to *E* might be optically activated.^10^ The polarization line was hindered when the chromophores started to re-align, indicating the beginning of the pillar domain motion at the nanoscale.^10^ Finally, re-aligned profiles at 75° to the *E* direction were observed after 360 s of light exposure, reaching a 90° configuration after an illumination time of 600 s (Fig. 2b).

This phenomenon might also be explained by the Maxwell theory on the electromagnetic nature of light,^27,28^ allowing the pillar domain motion (at the nano/sub-micro scale) to occur after the chromophore motion. This process was more likely due to the energy re-arrangement from the highest (*E*) to lowest energy direction (*B*), rather than the *trans*–*cis* isomerization at the molecular scale. This re-alignment was controllable as a function of time when other optical conditions were fixed, for example from 0° (*E*, parallel) → 75° → 90° (perpendicular) to the *E* direction (Fig. 2b).

Furthermore, nanobump-like structures were generated during the re-alignment, during which topographical fragmentations at a surface depth of 200 nm for each individual pillar were present after 480 s and 600 s of light stimulation (Fig. 2c). The maximum re-aligned angle was observed when the sub-structures were perpendicular to the *E* direction after 600 s of light exposure during elongation (Fig. 2d).

This suggested two possibilities for the photo-viscoelastic deformation point: (i) the 90° dynamic region for viscoelastic deformation and energy re-arrangement between *E* and *B* and (ii) an asymmetric internal force from *E* to *B* generated by light during this process.

Therefore, a macroscopic pillar motion could be achieved by adjusting light conditions to induce a larger asymmetric force.

The photoactivation and photo-viscoelastic processes were also recognized in both cases; within the first 30 s of exposure, the pillars’ height decreased from 359 ± 47 nm to 226 ± 38 nm (Fig. 2e) along with an increase in the structure's aspect ratio from 1.04 ± 0.03 to 1.96 ± 0.21 (*L*_max_/*L*_min_, Fig. 2f).

**Fig. 3:**
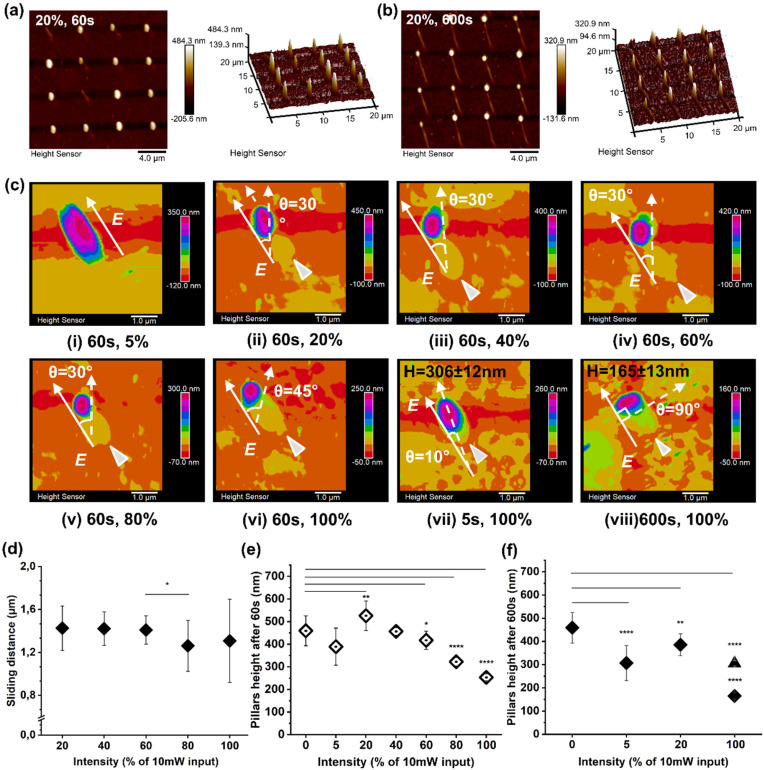
Pillar light response under different intensities. (a and b) Collective presentation of pDR1m pillar sliding under 20% intensity after 60 s (a) and 600 s (b) of stimulation. (c) AFM 3D visualization of pillar re-orientation and sliding under different intensities from 20% to 100% with different time periods as indicated in each graph. (d) Pillar sliding distance (tail-to-tail) quantification after 60 s under different light intensities, (e) pillar height after 60 s of stimulation under different laser intensities, (f) pillar height after 600 s of stimulation under different laser intensities (solid diamond symbol), and pillar height after 5 s of stimulation with 100% laser intensity (triangle symbol). Intensity is presented as the percentage of the total 10 mW input laser power.

Then, the influence of the illumination intensity on the deformation/motion process was investigated. Here, pillars sliding in the direction of the pillar polarization profile were observed for 20% laser intensity, featuring a controllable continuous sliding behavior from an illumination time of 60 s (Fig. 3a) to 600 s (Fig. 3b).

Further re-shaping, sliding, and rotation were observed and investigated while increasing the laser intensity from 5% to 100% (Fig. 3c). Here, after 60 s of illumination, the re-orientation angle to the *E* direction increased both in re-shaping and rotation, from 0° (under 5% laser intensity) to 30° for a laser intensity between 20% and 80%, and, finally a rotation of 45° was achieved for 100% laser intensity, as shown in Fig. 3c, i–vi. In the case of 20% laser intensity, the polymer material at the outer area of the pillar followed the 30° re-orientation while the pillar core remained stable (Fig. 3c, ii), suggesting a possible shear force generated by the light interaction as a result of photofluidization.^20^ In addition, the photofluidization phenomena at the pillar outer area induced by the intensive laser beam could reduce the friction between the vertical pillars and the planar substrate. Moreover, this process facilitated the sliding, whose direction was as for *E* and was not dependent on the re-orientation angle and illumination time. Finally, a full pillar rotation was obtained when higher laser beam intensities were applied (Fig. 3c, iii–vi).

Both the angle re-orientation and sliding were determined when the perpendicular direction to *E* was reached (100% laser intensity), allowing for identification of the yield point of the photo-viscoelastic deformation. The rotating direction of pillars also suggested the photofluidization-induced motion to be reversible between *E* and *B* re-arrangement directions (within the viscoelastic region). This offers the option to control both rotation and sliding behaviours, where a light-driven asymmetric force is required.

Subsequently, the photo-thermal effect had a major impact on the pillars’ deformation, with a height decrease from 306 ± 12 nm (after 5 s) to 165 ± 13 nm (after 600 s), as depicted in Fig. 3c, vii and viii.

Additionally, AFM measurements were carried out to quantify the average sliding distance under various light intensities (Fig. 3d).

Here, the friction between the pillars and the substrate was reduced by the light-driven force to allow the pillar to move, depending on the size of pillars and the nature of the substrate.^18^ Continuous photofluidization was therefore required to have a continuous asymmetric driving force to allow the pillars to slide further. As observed in the case of 20% intensity, an average sliding distance of 1.4 ± 0.2 μm was observed for an illumination time of 60 s up to 4.0 ± 0.4 μm for an illumination time of 600 s (Fig. 3d). A collective pillar array sliding behavior was also investigated by AFM (Fig. S7–9†).

The photofluidization process contributed to a height decrease during deformation as shown in Fig. 3e and f. However, a height increase was observed in the case of 20% intensity after 60 s of stimulation (Fig. 3e), suggesting that the pDR1m material can accumulate on the substrate upon laser stimulation. Moreover, the height decrease under 100% intensity after 600 s of illumination was attributed to the photo-induced plastic deformation (after the yield point), while sliding friction induced height loss in the viscoelastic region.^29^

**Fig. 4:**
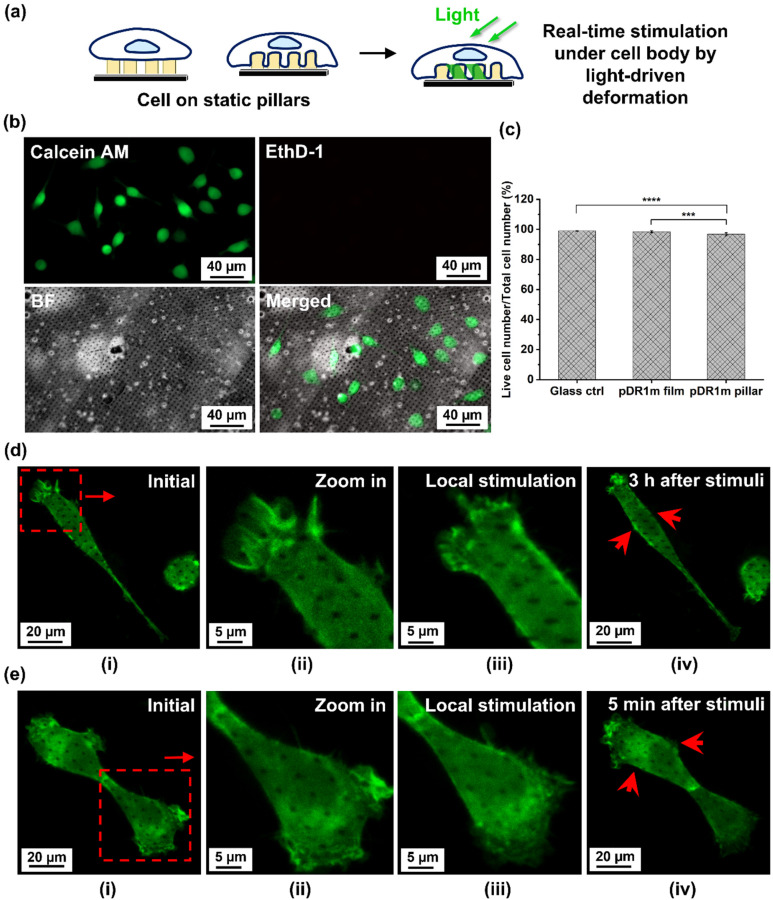
*In vitro* cellular evaluation of pDR1m pillars. (a) Schematic illustration of cell growth on static pillars and localized stimulation by light-driven deformation at single cell resolution. (b) Live/dead staining of wild type HT22 cells after 24-hour culture. Cells were presented with calcein AM staining (live, green), ethidium homodimer-1 staining (EthD-1, dead, red), bright field (BF) and merged imaging of all channels. Scale bar: 40 μm. (c) Live cell rate of HT22 cells after 24-hour culture; live cell rate was presented as the live cell number/total cell number × 100%. (d and e) LifeAct-GFP-HT22 cells on pDR1m pillar arrays: (i) initial status of whole HT22 cells, red squares show selected area for laser stimulation, scale bar: 20 μm; (ii) zoomed in view of the selected area, scale bar: 5 μm; (iii) selective stimulation and resulting shape changes of pillar arrays were observed for cells after 60 s of stimulation with a 555 nm laser at 5% intensity, scale bar: 5 μm; and (iv) cell response after stimulation at certain time points, red arrows indicate cytoskeleton motion after stimulation. Images were acquired after 72 h of LifeAct-GFP transfection.

Finally, we investigated how the controllable light-driven deformation of pDR1m pillars could also provide a local stimulation at the cellular level (Fig. 4a). Here, a cytotoxicity assay was performed with HT22 cells on static pDR1m pillar arrays after 1 day *in vitro* (DIVI), as shown in Fig. 4b and c and Fig. S10 and 11,† confirming the biocompatibility of the platform (96.8 ± 1.0% of living cells). To further characterize how local cytoskeleton stimulation could be achieved, live cell imaging was performed with HT22 transfected with LifeAct-GFP to identify actin filaments and their reshaping upon pillar deformation.

By illuminating pillars under the cell body, a localized stimulation was generated within one single cell. After 60 s of illumination, cell viability was maintained with visible live cytoskeleton motion in LifeAct-GFP-HT22 cells in the following 3 hours (Fig. 4d and e). Furthermore, cell motion was also monitored when stimulating the neighboring cell; the detection of a cytoskeleton response after 5 min stimulation of the neighboring cell (Fig. 4e) suggests the potential that interface signals could be transmitted from the platform to the target cell and the surrounding cells. However, we also point out that further systematic characterization of light-driven pillar deformation at the cell interface is still required to understand better the correlation between deformation and cell–cell communication. This includes studying parameters such as pillar motion, bending, and rotation, as well as correlating the effects of photon scattering under biological conditions.

## 3 Conclusions

The light-driven motion of pDR1m pillars at the micro–nano scale has been reported, and a photo-deformation model has been proposed, classifying the light-driven response into three mechanical regions. In this model, changes in pillar width are linked to the deformation process, as the width direction is perpendicular to *E*, the maximum extent that the pillars can reach. Width changes in low-intensity cases (5%) as a function of illumination time have been observed and discussed. In line with the general polymer material deformation mechanics, a three-stage photo-deformation model has been proposed for an azobenzene-based polymer in this work: photo-activation (photo-elastic deformation), photo-viscoelastic deformation (including both photo-elastic and photo-plastic deformation), and photothermal deformation (photo-plastic deformation). Photo-viscoelastic pillar deformation involves two components: chromophore motion and pillar domain motion. Chromophore motion is distinguished by a remaining polarization line and decreased width, with molecular motion concluding as the polarization line disappears. Subsequently, domain motion at the nanoscale begins; this process allows for the formation of substructures with a re-aligned pattern, achieving a maximum 90° deforming angle from *E* to *B*. The appearance of a second line perpendicular to *E*, generating topographical nanobumps, marks the end of photo-viscoelastic deformation. While additional details and mechanical evidence at the nanoscale and molecular scale are needed to further support this model, this work demonstrates its potential and provides tools for multidimensional nanomanipulation and biointerfacing. By creating an active and responsive interface, this work suggests how stimuli-responsive materials could be employed in bioengineering and the design of biomimetic systems.

## 4 Experimental section

### 4.1 Pillar masters and mold fabrication

Masters were fabricated by 2-photon lithography (Nanoscribe Photonic Professional GT system, Nanoscribe GmbH, Germany) in a DiLL configuration with a ×63 objective. Fused silica substrates – 25 × 25 mm^2^ with a thickness of 0.7 mm – were used. Then, the samples were immersed in mr-Dev 600 (Microresist Technology GmbH) for 10 min, and then in isopropanol (IPA, Merck, Germany) for 10 min for pattern development. Pillar arrays were designed using AutoCAD software (AutoDesk, USA), and then exported to an .STL file and further converted using DeScribe software (Nanoscribe GmbH, Germany). Pillar arrays were fabricated in a uniform parameter with 1 μm in height, 1 μm in diameter, and 4 μm pitch (edge-to-edge) (H1D1P4, Fig. S1†). Ip-Dip resist (Nanoscribe GmbH, Germany) was used as the printing material that was patterned with an output power of 45 mW and a writing speed of 10 000 μm s^−1^.

For polydimethylsiloxane (PDMS) mold fabrication, PDMS pre-solution (Sylgard 184 Silicone Elastomer Kit, 10 : 1 w/w related to elastomers: curing agent, purchased from Dow Chemical Company, USA) was gently added on the photoresist pillar array. The sample was transferred to an 80 °C oven for 4 hours to complete polymerization, and then the PDMS mold was peeled off of the glasses.

### 4.2 pDR1m film fabrication and pDR1m pillar imprinting

Poly(disperse red 1 methacrylate) (pDR1m, CAS: 139096-37-0, Sigma-Aldrich Chemie GmbH) was dissolved in chloroform (Sigma-Aldrich Chemie GmbH) at 3.5 w/v% concentration. 15 μL of the prepared pDR1m solution was applied to a cover glass (*d* = 12 mm) using a spin coater (Laurell Technologies, WS-650Mz-23NPPB) at 2500 rpm for 40 seconds (×3). The glass was washed with ethanol (×3) and deionized water (×3) before use. The coated sample was air dried in a fume hood. For imprinting pDR1m pillar arrays, the PDMS mold was covered with the above prepared pDR1m film on the top, and then kept in an oven at 150 °C overnight to allow pDR1m to slowly melt into the PDMS mold. Pillar arrays were obtained by peeling off the PDMS mold from the cover glass.

### 4.3 Confocal laser-based pillar shaping

A 555 nm laser from an LSM700 confocal microscope (Zeiss GmbH, Germany) was applied to shape the pillars and bright field images were obtained under a 639 nm laser. Image size was set at 45 μm × 45 μm with the pixel size at 0.09 μm. Shape change with different illumination times was tracked from 0 to 600 s. Pillars were flipped on the sample stage to face the laser input (as a confocal laser goes from bottom-up), held by a glass slide. Different intensities of the 555 nm laser were investigated from 5% to 100% of the total LSM700 confocal input power (10 mW). All laser power percentages in this work are based on a total input power of 10 mW, unless otherwise stated. The actual laser output intensity and the output power density that fall onto the samples and to the selected stimulation area are presented in Table S1.†

### 4.4 Atomic force microscopy

The 3D structures of the shaped pillars were visualized by atomic force microscopy (Bruker-ICON, USA) in air with a ScanAsyst-fluid cantilever (Bruker, USA) under ScanAsyst mode. All the presented data were obtained under a 1 Hz scan rate. Data were analyzed and presented using NanoScope Analysis software. Quantification of pillar width, height, and moving distance change was performed using a patterned sample analysis tool on the sphere/ellipse feature type. All 3D views of the pillar structures were presented at a *Z* axis aspect ratio of 0.3.

### 4.5 Optical stimulation and analysis

Optical motion of pDR1m pillars was triggered by using a Zeiss LSM700 confocal microscope under bright field. A time series method under continuous illumination with a 2 s frame step was applied to collect the data; 300 frames were collected with a total time of 600 s. Different laser intensities (5%, 20% and 100%) were applied to investigate and visualize the light-driven motion. The video presented was compressed four times on a time scale from the original 600 s to 150 s.

### 4.6 Sample cleaning and polylysine coating for cell experiments

Poly(disperse red 1 methacrylate) pillar arrays (H1D1P4, 1 mm × 1 mm) on cover glasses (*d*: 12 mm) were chosen for the biocompatibility assay. All samples were gently cleaned with acetone to remove pRD1m residues and to define a culturing area (3 mm × 3 mm) on glass. All samples (including glass controls) were sterilized under UV light (365 nm) for 1 hour. To improve cell attachment, substrates were immersed in 0.01 wt% poly-l-lysine (Merck, Germany) solution in phosphate buffered saline (PBS, pH 7.4, Gibco™, USA) buffer (600 μL) at 37 °C overnight. Unreacted polylysine was washed away using PBS (1×, pH 7.4, Gibco™, USA) three times before cell plating.

### 4.7 Biocompatibility evaluation with HT22 cells

Dulbecco's modified Eagle's medium/nutrient mixture F-12 Ham (DMEM/F12, D6421, Thermo Fisher Scientific, USA) medium was used for cell culture, supplemented with 10% fetal bovine serum (FBS, Gibco™, USA), 1% l-glutamine (CF = 2 mM) (Life Technologies, USA) and 1% penicillin–streptomycin (PS, Sigma-Aldrich, USA) as a basal cell culture medium in this work. 50k HT-22 mouse hippocampal neuronal cells (HT22, Sigma-Aldrich, SCC129) were seeded on each glass; live/dead imaging and counting were performed after 24-hour culture to evaluate the cytotoxicity of the samples. The LIVE/DEAD™ viability/cytotoxicity kit for mammalian cells (Invitrogen™, L3224, Thermo Fisher Scientific, USA) was used for live/dead staining, and calcein AM and ethidium homodimer-1 from the kit were diluted to a final working concentration of 1 : 1000 with PBS, and 300 μL of the working solution was added to each sample, followed by 20 min of incubation at 37 °C. Images were acquired using an Axio Vario microscope (Zeiss, Germany) with a ×20 objective at ×1.6 zoom magnification. Bright field images were taken before live/dead staining. Both live cells and dead cells were counted using ImageJ for statistical analysis. Data were presented in live cell number/total cell number as percentage.

### 4.8 DNA extraction

The pCMV-LifeAct®-TagGFP2 plasmid (cat. no. 60101) was ordered from ibidi®, Germany. DNA extraction was performed using the Plasmid Plus Midi Kit (Qiagen). The protocol was followed according to the manufacturer's instructions and DNA was eluted in 100 μl of TE buffer. Plasmid DNA was stored at −20 °C. DNA content was quantified by absorbance with the Nanodrop facility (Thermo Fisher Scientific, Germany).

### 4.9 Lipofection of HT22 cells

pDR1m arrays on 12 mm cover glasses were placed in a 24-well plate and HT22 cells were seeded onto each sample with a final density of 10.5 k cm^−2^; the cell culture medium was maintained at 1.2 mL per well. Lipofection was applied with lipofectamine™ 3000 (Thermo Fisher Scientific, USA) after 24-hour culture without changing the medium. Two tubes (A and B) were needed for lipofection. In tube A, 1 μL of P3000 was added into 25 μL of Opti-MEM™ (Thermo Fisher Scientific, USA), and then 300 ng of DNA was gently added for each sample. In tube B, 0.75 μL of lipofectamine was added into 25 μL of Opti-MEM® for each sample. Tubes A and B were mixed gently for 15 min at room temperature (∼21 °C), and then the mixture was added to each well dropwise. The plate was gently shaken before returning to the cell culture incubator to achieve homogeneous lipofection. Live cell imaging was carried out after 48 hours of lipofection.

### 4.10 Live cell imaging

Lifeact® transfected HT22 cells on substrates were transferred to 35 mm confocal Petri dishes (glass bottom) and the medium was replaced by 1.2 mL basal cell culture medium (medium recipe see 4.7). Samples were placed in an Okolab stage top incubator (Okolab, Italy) at 37 °C. A Zeiss LSM700 confocal microscope was used for stimulating and imaging. For stimulation, pillars were shaped with a 555 nm LSM700 confocal laser which came from under the cells. 5% of the total 10 mW input intensity was used in this session at a frame size of 45 μm × 45 μm with a pixel size of 0.09 μm. 60 s illumination was applied to the area of interest. After pillar reshaping, fluorescence images of Lifeact® transfected HT22 cells were acquired under 488 nm excitation and 518 nm emission.

### 4.11 Statistical analysis

All the results presented in this work were collected from at least three independent experiments with three individual replicates. Data quantification variance was analyzed by the one-way ANOVA method with OriginPro® 2022b (OriginLab, USA); significant difference was considered when the *p* value < 0.05. The statistical difference level was labeled when *p* < 0.05 (*), *p* < 0.01 (**), *p* < 0.001 (***) and *p* < 0.0001 (****).

## Author contributions

In this work, Ziyu Gao contributed to the experimental design, data collection, analysis, and manuscript writing. Valeria Criscuolo contributed to 2-photon lithography for master fabrication. Fabio Formiggini provided technical support on the optical setup and AFM. Ilaria De Martino, Daniela Perna and Velia Siciliano supported the molecular biology experiments. Francesca Santoro contributed to project design, data discussion, manuscript revision and funding acquisition.

## Conflicts of interest

There are no conflicts to declare.

## Supplementary Material

NR-017-D4NR04659E-s001

## Data Availability

Data for this article will be made available upon request to the authors.
