# Supplementary material for: Micro–nano scale motion control of light-driven morphing pillars for biointerfacing
Source: Nanoscale. 2025 Jun 10;17(27):16493–502. doi: 10.1039/d4nr04659e (PMC12188518; doi:10.1039/d4nr04659e)
Supplement: NR-017-D4NR04659E-s001 [file NR-017-D4NR04659E-s001.pdf]

Supplementary Information (SI) for Nanoscale.
This journal is © The Royal Society of Chemistry 2025

Supporting Information

Micro-nano scale motion control of light-driven morphing pillars for biointerfacing

Ziyu Gao [1,2,3,#], Valeria Criscuolo [1,2,3], Fabio Formiggini [1], Ilaria De Martino [1], Daniela Perna [1], Velia Siciliano [1], Francesca Santoro [1,2,3,*]

[a] Center for Advanced Biomaterials for Health Care, Istituto Italiano di Tecnologia, Naples, Italy.
[b] Institute of Biological Information Processing – Bioelectronics, IBI-3, Forschungszentrum Jülich, Jülich, Germany.
[c] Neuroelectronic Interfaces, RWTH Aachen, Aachen, Germany.

[#] Current address: Research group of Cell-sensor Hybrids and Neuroimplants, Institute of Materials in Electrical Engineering 1, Faculty of Electrical Engineering and Information Technology, RWTH Aachen University, 52074 Aachen, Germany.

*Correspondence to: f.santoro@fz-juelich.de

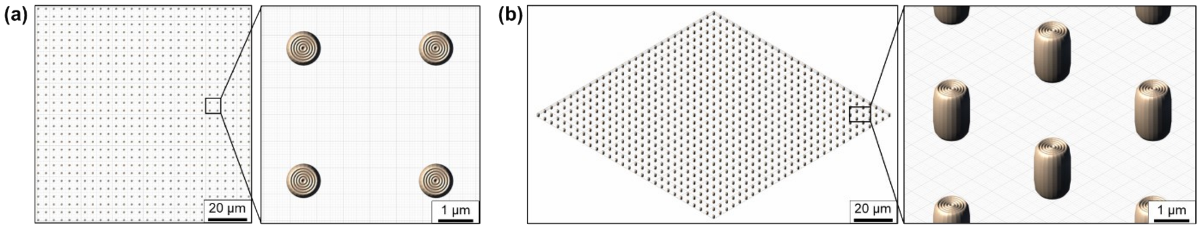

Figure S1. Describe render of micropillar array design: top view (a) and 45° tilted view (b) of H1D1P4 array of area 272 µm².

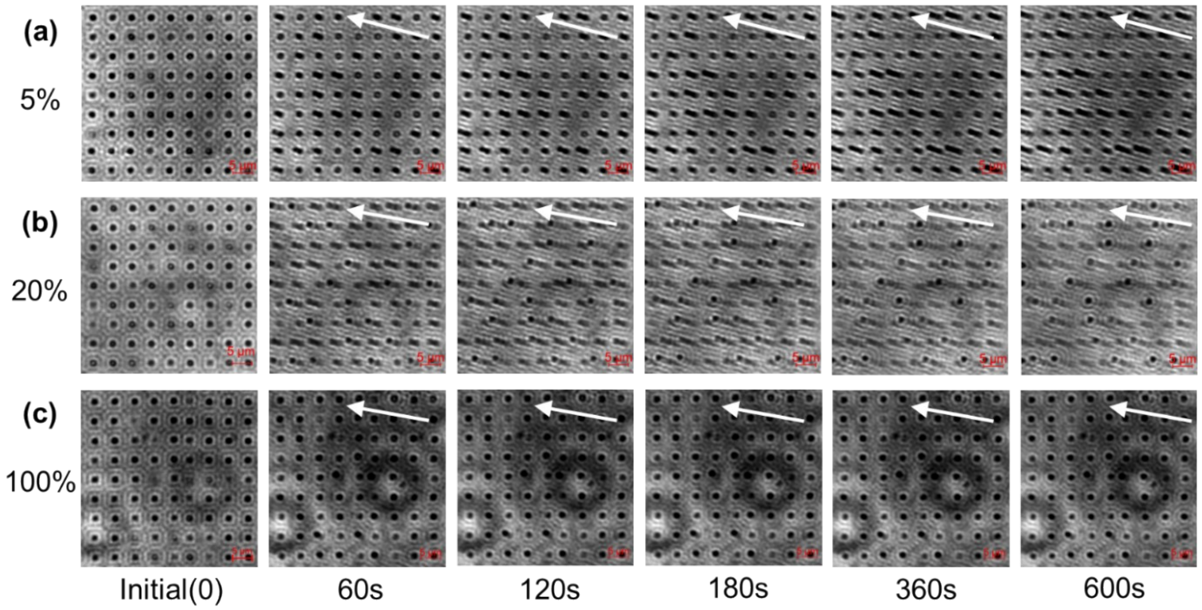

Figure S2. Optical images of time-dependent light-driven deformation on pDR1m pillar arrays. Deformation under different laser intensities, from 5% (a), to 20% (b), to 100% (c) of total 10 mW laser input power. Deformation after different illumination time from 0 up to 600 s was tracked (from left to right column). White arrows indicate pillar morphing direction (polarization). Scale bar: 5 µm.

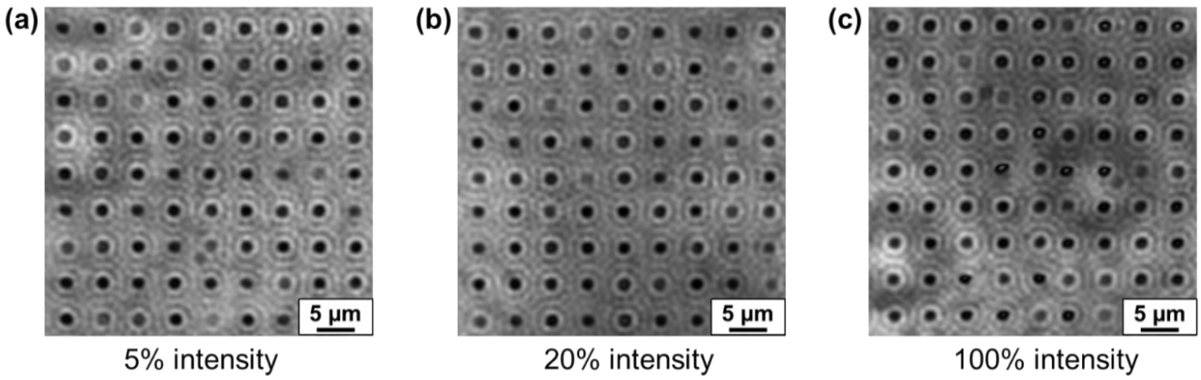

Figure S3. Optical video record of time-dependent light-driven deformation on pDR1m pillar arrays. Deformation under different laser intensities, from 5% (a, Media 1), to 20% (b, Media 2) and 100% (c, Media 3) of total 10 mW laser input power. Scale bar: 5 µm.

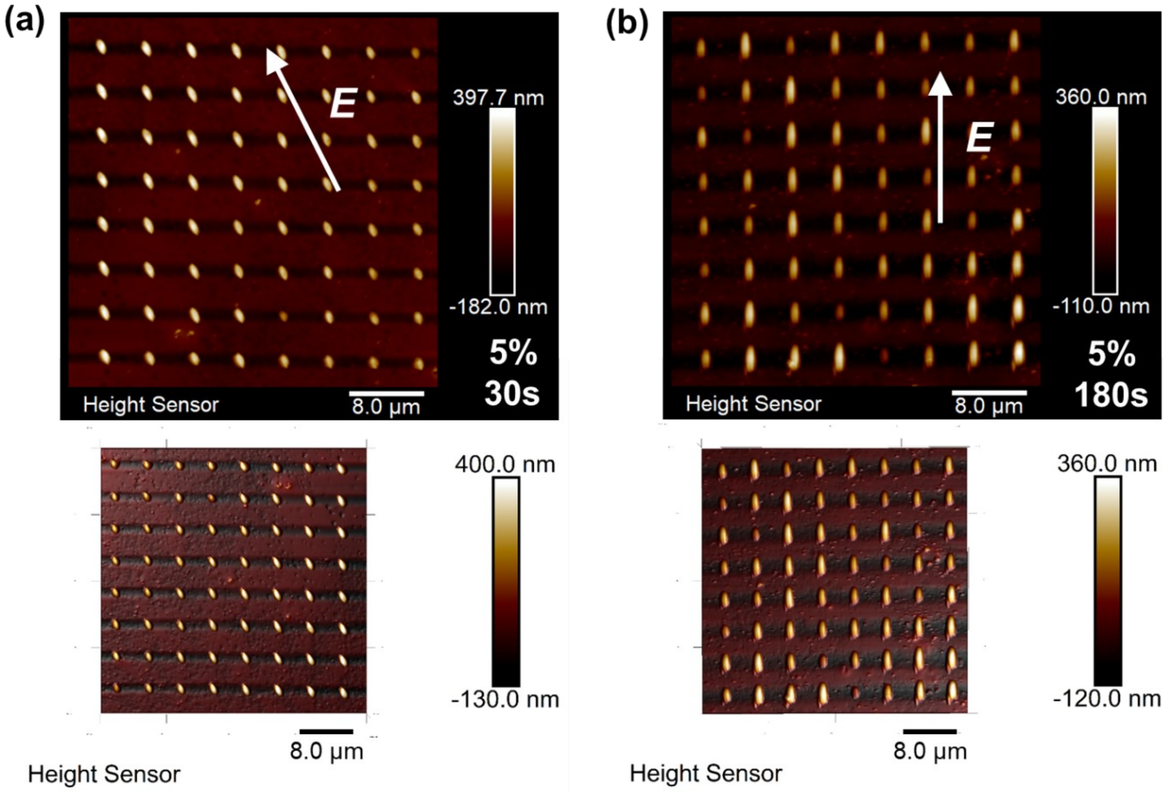

Figure S4. Morphology track of pDR1m pillars shaping under 555 nm laser with 5% intensity after 30 s (a) and (b) 180 s illumination time, at different exposure direction. Scale bar: 8 µm.

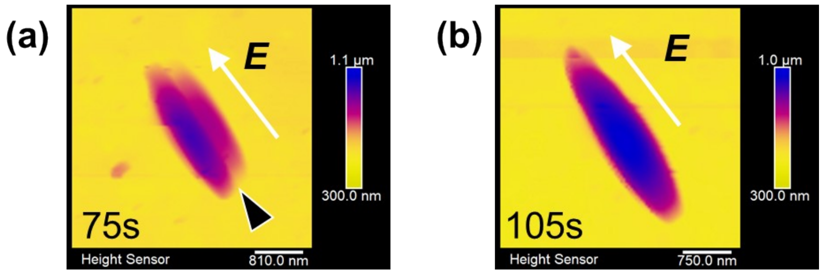

Figure S5. Morphology track of pDR1m pillars shaping under 555 nm laser with 5% intensity after 75 s (a) and (b) 105 s illumination time. E indicates polarization direction. Laser intensity presented in percent of total 10 mW input power. Scale bar: 750 nm.

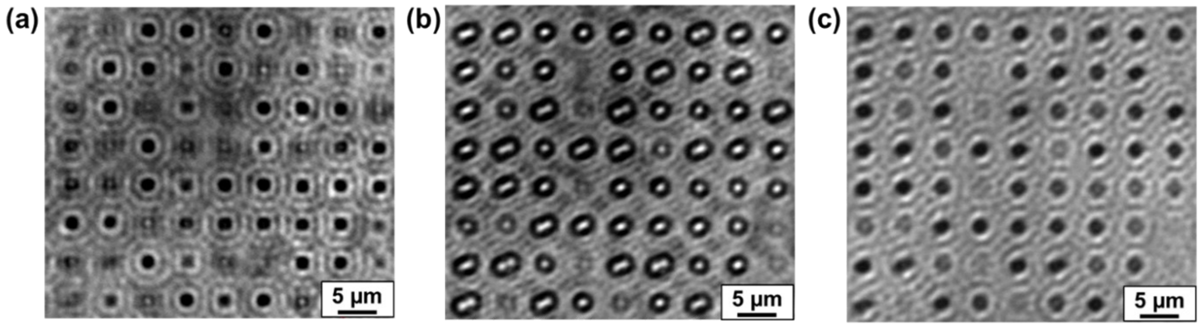

Figure S6. Optical images of time-dependent recovery test on pillar arrays. (a) initial pillar arrays, (b) illuminated by 555 nm laser beam under 5% intensity after 30 s, (c) after 2-hour recovery in air. Scale bar: 5 µm.

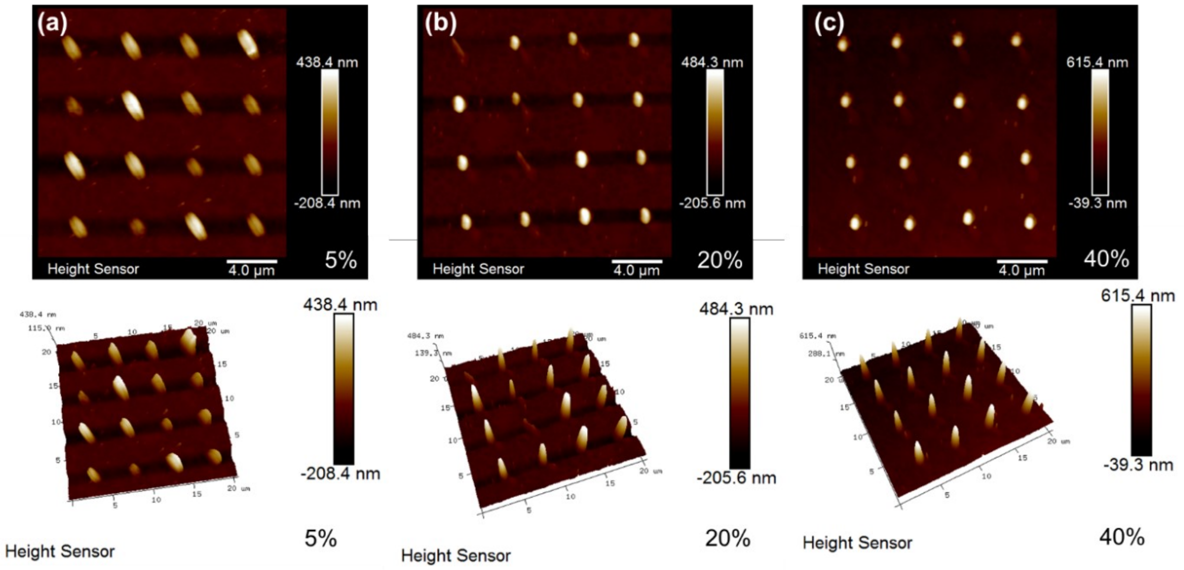

Figure S7. AFM visualized sliding motion of pillars under 5% (a), 20% (b), and 40% (c) intensity, after 60 s stimulation. Total laser input 10 mW.

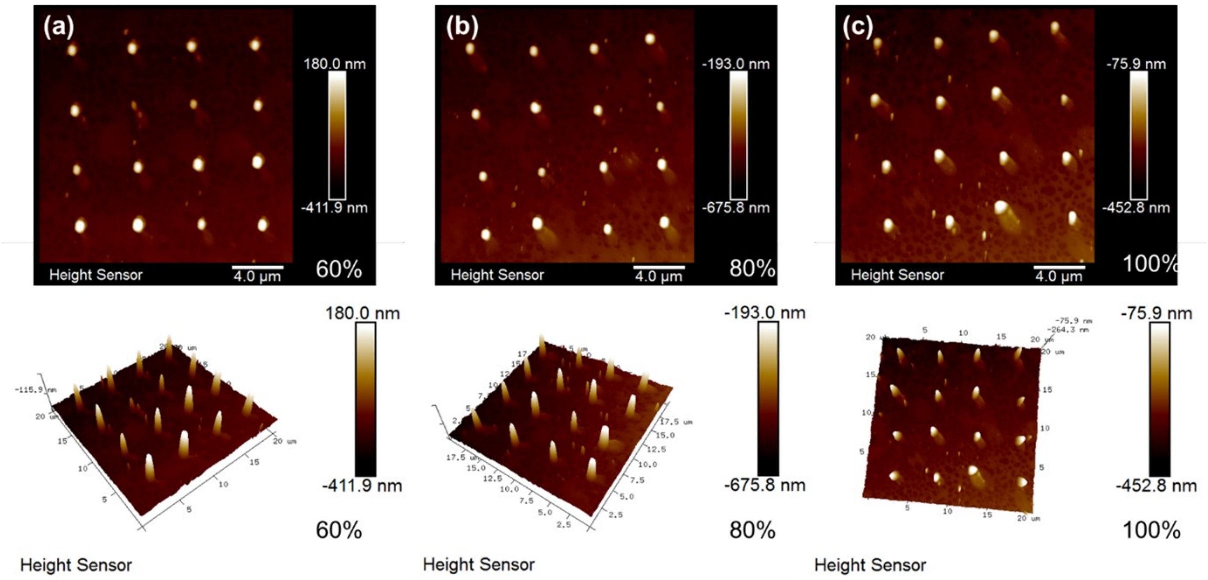

Figure S8. AFM visualized sliding motion of pillars under 60% (a), 80% (b), and 100% (c) intensity, after 60 s stimulation. Total laser input 10 mW.

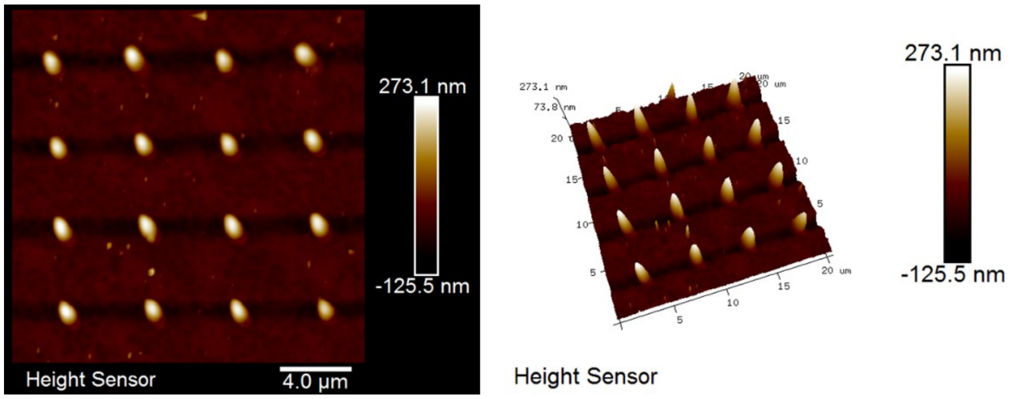

Figure S9. AFM visualized sliding motion of pillars after 5 s stimulation under 100% intensity, 2D view (left) and 3D view (right). Total laser input 10 mW.

Table S1. Actual output intensity of 555 nm laser at different percents which were applied in this work. Measured at objective panel by Thorlabs power meter mod. PM100D with sensor mod. S121C. The power density was estimated based on the field of view from Obiective 10x/0.3 and the selected frame size that used in this work for light stimulation.

Input laser power [%] [a]	Output laser power [mW]	Output intensity (mW/mm^2) (Estimated for Objective 10×/0.3)	Stimulating intensity (mW/mm^2) (Estimated for stimulating area, 45 μm^2)
5	0.13	0.026	64
10	0.26	0.053	128
20	0.53	0.108	262
40	1.07	0.218	528
60	1.62	0.330	800
70	1.89	0.385	933
80	2.17	0.442	1072
90	2.44	0.497	1205
100	2.70	0.550	1333

[a] Percent of total 10 mW input power.

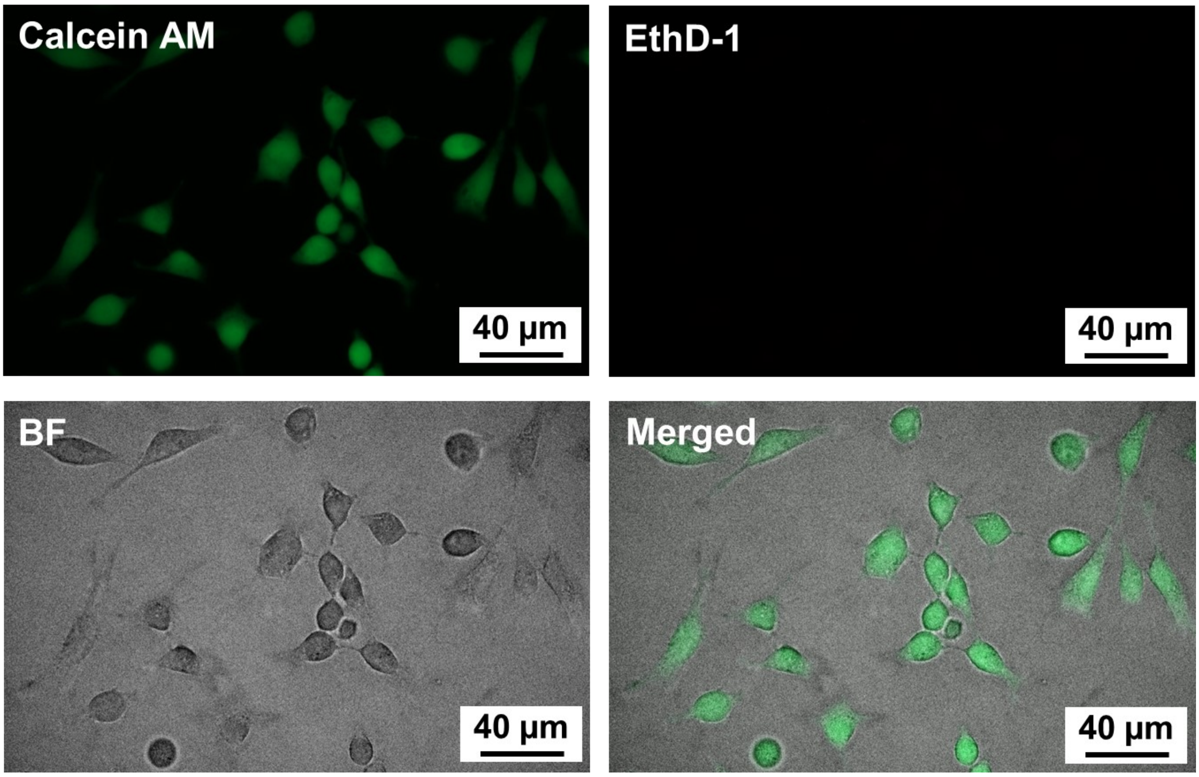

Figure S10. Live/Dead staining of HT22 on cover glasses after 24-hour culture. Cells were presented with Calcein AM staining (live, green), Ethidium homodimer-1 staining (EthD-1, dead, red), bright field (BF) and merged imaged of all channels. Scale bar: 40 µm.

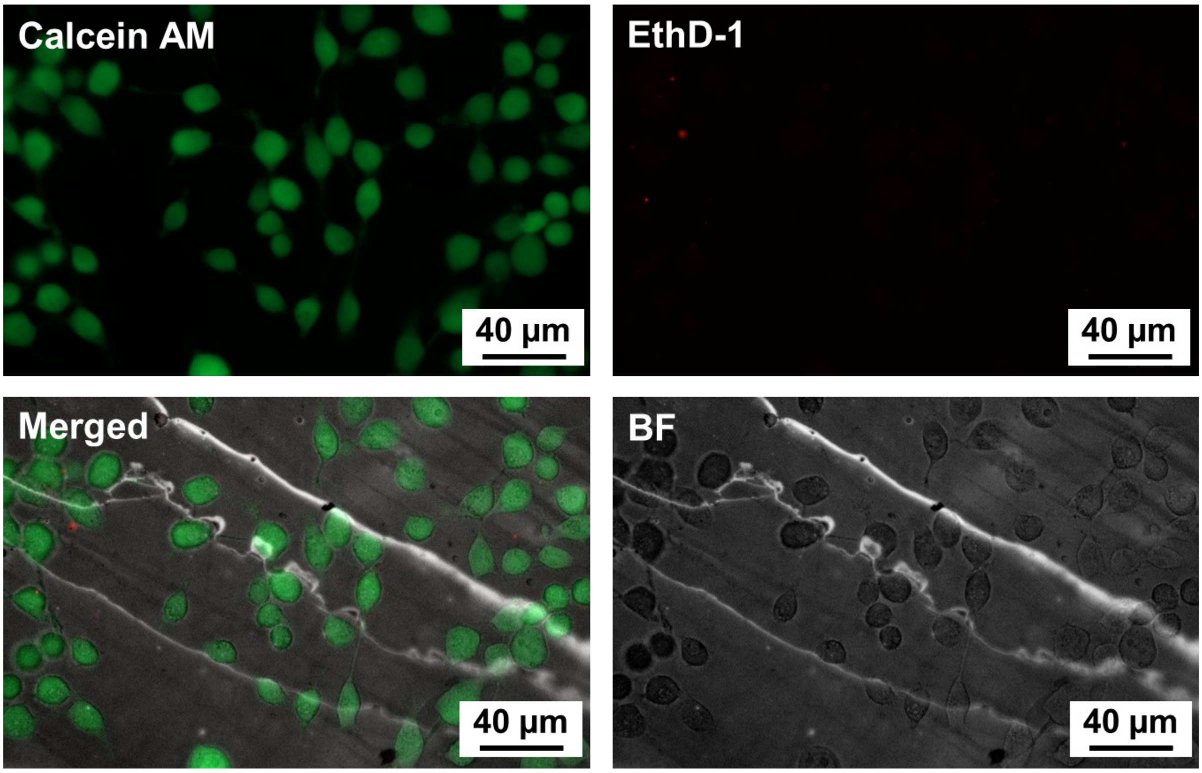

Figure S 11. Live/Dead staining of HT22 on pDR1m film after 24-hour culture. Cells were presented with Calcein AM staining (live, green), Ethidium homodimer-1 staining (EthD-1, dead, red), bright field (BF) and merged images of all channels. Scale bar: 40 μm.
